# Circadian onset and prognosis of myocardial infarction with non-obstructive coronary arteries (MINOCA)

**DOI:** 10.1371/journal.pone.0216073

**Published:** 2019-04-25

**Authors:** Anna M. Nordenskjöld, Kai M. Eggers, Tomas Jernberg, Moman A. Mohammad, David Erlinge, Bertil Lindahl

**Affiliations:** 1 Department of Cardiology, Faculty of Medicine and Health, Örebro University, Örebro, Sweden; 2 Department of Medical Sciences and Uppsala Clinical Research Center, Uppsala University, Uppsala, Sweden; 3 Department of Clinical Sciences, Danderyd Hospital, Karolinska Institute, Stockholm, Sweden; 4 Department of Cardiology, Clinical Sciences, Lund University, Skane University Hospital, Lund, Sweden; University of Messina, ITALY

## Abstract

**Background:**

Many acute cardiovascular events such as myocardial infarction (MI) follow circadian rhythms. Myocardial infarction with non-obstructive coronary arteries (MINOCA) is a newly noticed entity with limited data on onset pattern and its impact on prognosis.

**Material and methods:**

In this observational study of Swedish MINOCA patients registered in the SWEDEHEART registry between 2003–2013 and followed until December 2013 we identified 9,092 unique patients with MINOCA out of 199,163 MI admissions in total. Incidence rate ratios (IRR) were calculated for whole hours, parts of the day, weekdays, months, seasons and major holidays.

**Results:**

The mean age was 65.5 years, 62.0% were women and 16.6% presented with STEMI. The risk for MINOCA proved to be most common in the morning (IRR = 1.70, 95% CI [1.63–1.84]) with a peak at 08.00 AM (IRR = 2.25, 95% CI [1.96–2.59]) and on Mondays (IRR = 1.28, 95% CI [1.18–1.38]). No altered risk was detected during the different seasons, the Christmas and New Year holidays or the Swedish Midsummer festivities. There was no association between time of onset of MINOCA and short- or long-term prognosis.

**Conclusion:**

The onset of MINOCA shows a circadian and circaseptan variation with increased risk at early mornings and Mondays, similar to previous studies on all MI, suggesting stress related triggering. However, during holidays were traditional MI increase, we did not see any increase for MINOCA. No association was detected between time of onset and prognosis, indicating that the underlying pathological mechanisms of MINOCA and the quality of care are similar at different times of onset but triggering mechanism may be more active early mornings and Mondays.

## Introduction

A majority of acute cardiovascular events such as myocardial infarction (MI) [1–6], sudden cardiac death [1, 5], rupture of aortic aneurysms [5], ischemic and hemorrhagic stroke [5] tend to be more common early mornings.

In addition to a circadian rhythm, the onset of MI has a weekly rhythm [6–8] with higher rates on Mondays and a seasonal rhythm [7, 9] with higher rates of onset during winter. Increased rates of MI have also been noted during Christmas and New Year holidays [6, 7].

Myocardial infarction with non-obstructive coronary arteries (MINOCA) is a newly noticed clinically important entity with higher incidence than previously recognized. Patients with MINOCA as compared to patients with MI with obstructive coronary arteries (MI-CAD) tend to be younger [10, 11] and more often female [10, 11]. The underlying pathophysiological mechanisms are largely unknown but several mechanisms have been suggested; plaque disruption, coronary artery spasm, coronary thromboembolism, coronary dissection, microvascular dysfunction or type 2 MI [12, 13].

We lack information on triggering factors specific for MINOCA and on possible circadian or seasonal rhythms related to onset or prognosis. The aims of the present study were therefore twofold; firstly, to investigate the presence of any circadian rhythm (day-and night, week, month, season) and association with major holidays (Christmas, New Year and the Swedish Midsummer festivities) to the onset of MINOCA and secondly, to investigate if the prognosis for MINOCA patients varies after different times of onset.

## Materials and methods

### Study population

**Fig 1:**
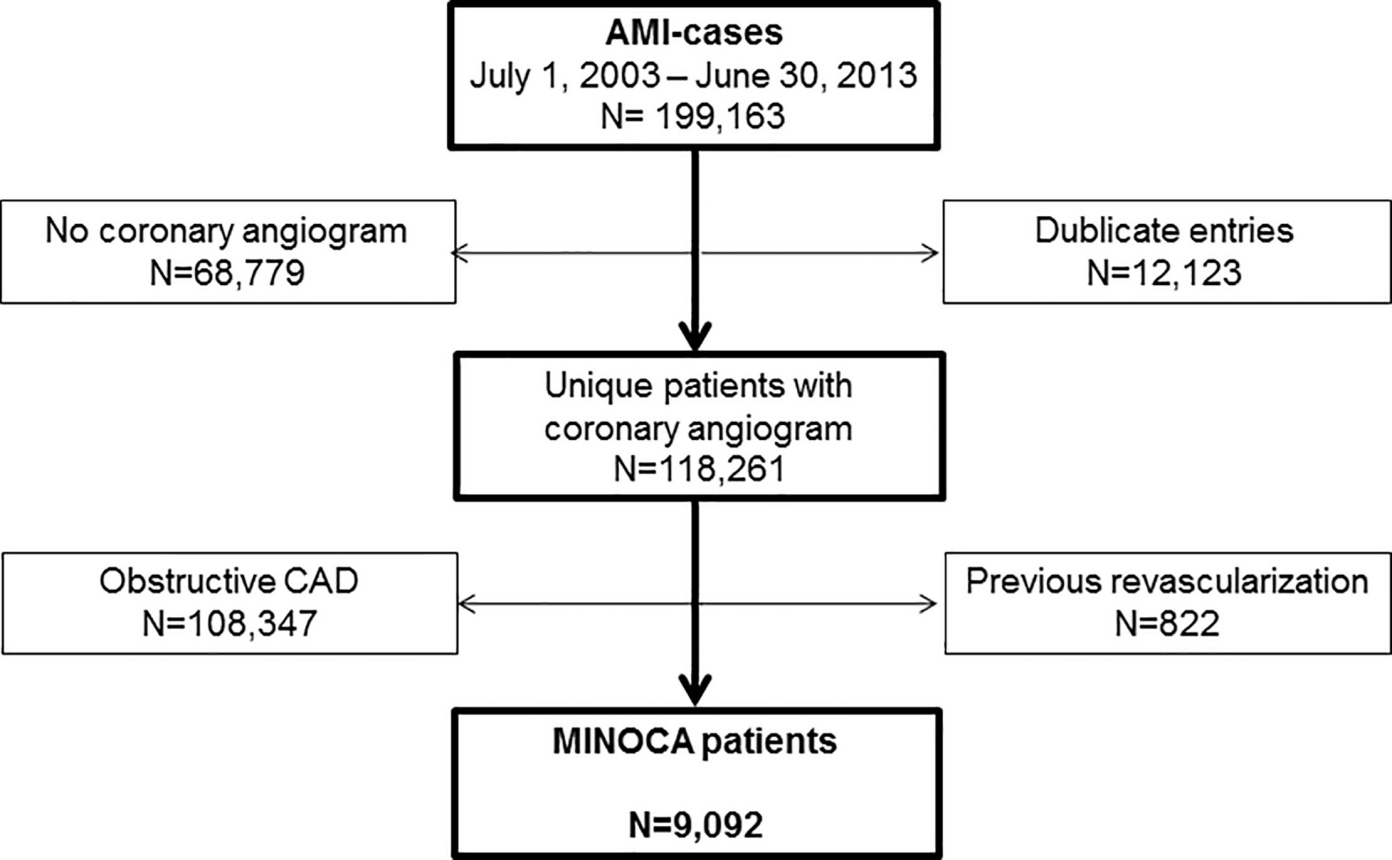
The study population.

The MINOCA population assessed in the present study has been presented before [10, 14]. Briefly, the study cohort was extracted from SWEDEHEART, a nationwide registry enrolling consecutive patients admitted to specialized care units due to suspected acute coronary syndrome. A total of 9,092 singular MINOCA patients were extracted out of 199,163 admissions due to acute MI recorded between July 2003 and June 2013 (Fig 1).

Patients were identified as having MINOCA if the discharge diagnosis was acute MI (International Classification of Diseases, 10th Reversion code: I21-I22) and a coronary angiography performed during hospitalization did not show a diameter stenosis of 50% or more. Patients with a discharge diagnosis of Takotsubo syndrome (ICD 10 code I42.8) or myocarditis (ICD 10 code I40) were not included.

The SWEDEHEART registry contained data on baseline characteristics, self-reported onset time and date for the current symptoms, biochemical markers, ECG changes, left ventricular ejection fraction (LVEF), coronary angiography results, medical and invasive treatment and outcome (see http://www.swedeheart.se for details). Monitors from participating centers validate the data entered into the SWEDEHEART, and over the years there has been a >95% agreement between data in the registry and in the hospital records [15].

### Coronary angiography

Data regarding the coronary angiographies were extracted from the SWEDHEART registry [15], in which the angiograms are interpreted by the local interventionalist. The degree of narrowing of diameters is categorized as <50%, 50–69%, 70–99% or 100% (occlusion). Angiographic data are subject to routine monitoring as part of the SWEDEHEART standard operations. The Classification of any stenosis is done according to the ACC/AHA Task Force [16].

### Outcome definitions

To study circadian rhythm, the time points of the first symptoms indicating MINOCA were: a) rounded to full hours and b) divided into four diurnal periods of time; 00:00–05:59 (night), 06:00–11:59 (morning), 12:00–17:59 (afternoon) and 18:00–23:59 hours (evening).

The dates of symptoms indicating MINOCA were transformed into: a) weekdays b) months (January-December) c) seasons; winter (December-January-February), spring (March-April-May), summer (June-July-August) and autumn (September-October-November) and d) major holidays. The holidays investigated were the Christmas and New Year holidays (December 20 –January 3) compared to the 2 weeks preceding and following (December 5–19 and January 4–18), Christmas Eve and Christmas Day (December 24–25) compared to corresponding days prior and after the holiday (December 10–11 and January 7–8) and the Swedish Midsummer festivities (June 19–26 June) compared to one week prior and one week after (June 13–18 and June 27 –July 4).

Short term prognosis is defined as death within 30 days of the MINOCA. Long term prognosis is defined by the pre-specified combined endpoint of major adverse cardiovascular events (MACE) consisting of all-cause death, new MI and hospitalization for heart failure or ischemic stroke. Due to low power we refrained from investigating the prognosis associated with onset of symptoms during the holidays.

### Subgroups

Pre-specified subgroup analyses were assessed for all periods of interest and for the association with short- and long-term prognosis. The subgroups were based on gender, age and ECG showing ST-elevation (STE-MINOCA) or Non ST-elevation (NSTE-MINOCA).

### Follow-up

Follow-up data on patient outcome was acquired by merging data from SWEDEHEART with data from the mandatory Swedish Patient Registry (hospitalization dates and discharge diagnoses based on International Classification of Diseases 10 codes [ICD-10]) and the Swedish Cause of Death Registry, both possessed by the Swedish Board of Health and Welfare. Patients were followed for events until occurrence of death or December 31, 2013 with a mean follow-up of 52 months.

The study was approved by the Regional Ethical Review Board in Stockholm (2012/60-31/2). All patients in Sweden must be informed about their participation in registries and their right to decline participation. However, according to the Swedish law there is no need for written consent.

### Statistics

The incidence rate ratios (IRR) for MINOCA were calculated from the onset of symptoms by hour, time period (night, morning, afternoon, evening), day of the week, month, season (winter, spring, summer, autumn), the Christmas and New Year holiday, the Christmas Eve and Christmas Day and the Midsummer festivities by applying a Poisson regression model. Likelihood Ratio Chi-Square test from the Poisson regression model was used as an overall test to detect any difference, in respect to the number of events any given time point, between the pre-specified time points/periods. The Bonferroni-Holm method was used to control for errors owing to multiple testing in all analysis of IRR at different time periods.

In order to identify time and periods associated with the short-term prognosis binary logistic regression analyses were performed. In order to identify time and periods associated with long-term prognosis univariable and multivariable Cox regression analyses were performed. Models containing one of the different pre-specified time points/periods together with known risk factors for death/ MACE (gender, diabetes, age group, smoking, LDL-cholesterol group) were used. Visual assessment of Cox regression log minus log plots were used to find any differences associated with follow-up time. Hazard ratio (HR) with corresponding 95% confidence intervals (CI) are reported.

Age and LDL-cholesterol were regarded both as continuous variables and categorical variables. For age two different categorical variables were created; a) dichotomous: Age ≥65 years and <65 years and b) five different age groups; <50 years, ≥50 and <60 years, ≥60 and <70 years, ≥70 and <80 years and ≥80 years. LDL-cholesterol was used both as a continuous variable and a categorical variable. Three different groups were created; <1.8 mmol/L, ≥1.8 to <3 mmol/L and ≥3 mmol/L.

The Predictive Analytical SoftWare (PASW statistics 17.03) program (SPSS Inc, Chicago, IL, USA) was used for all analyses. In all tests, a 2-sided p- value <0.05 was considered statistically significant.

## Results

Table 1 present the baseline clinical characteristics which are previously reported [10, 14]. Women make up 62.0% of the cohort and the mean age was 65.5 years. Known risk factors were common among the patients and 13.6% suffered from diabetes, 48.2% had hypertension and 51.7% were current or previous smokers. A total of 1483 patients (16.6%) presented with STE-MINOCA at admission and the baseline characteristics are stratified according to the ECG finding (Table 1). A total of 31.2% of the patients were employed; 66.5% of patients <65 years and 3.9% of patients ≥65 years, respectively.

**Table 1:** Baseline characteristics. Characteristics for all MINOCA and divided according to the ECG at presentation (STE-MINOCA vs NSTE-MINOCA).

	All MINOCA	STE-MINOCA	NSTE-MINOCA	p-value*
Total, n	9,092	1,483	7,609	
**Demographics**				
Female (%)	62.0	59.8	62.5	0.054
Age, y (±SD)	65.5±11.5	65.8±12.8	65.4±11.2	0.258
**Risk factors (%)**				
Smoking				
Current	19.8	24.6	18.8	<0.001
Previous	31.9	27.6	32.8	
Diabetes	13.6	10.0	14.3	<0.001
Hypertension	48.2	44.2	49.0	0.001
Medical history, %				
Cancer	2.0	2.0	2.0	0.915
COPD	8.3	7.8	8.4	0.433
Dementia	0.2	0.4	0.2	0.071
MI	4.6	4.5	4.6	0.943
PVD	1.9	1.4	2.0	0.159
Stroke	5.4	5.3	5.5	0.828
**Laboratory findings**				
Creatinine, μmol/L (±SD)	80.8±39.3	81.5±39.6	80.6±39.2	0.423
CRP mg/L (IQR)	5.0 (3.0–10.0)	6.0 (3.0–15.0)	5.0 (2.7–10.0)	<0.001
LDL cholesterol, mmol/L (±SD)	3.0±1.0	2.9±1.0	3.1±1.0	<0.001
Total cholesterol, mmol/L (±SD)	5.1±1.2	5.0±1.1	5.1±1.2	<0.001
LVEF (%)				
≥50%	54.9	44.8	56.9	<0.001
40–49%	12.4	16.7	11.6	
30–39%	6.8	10.9	6.1	
<30%	3.2	4.2	3.0	
Unknown	22.6	23.5	22.5	
**Medication at admission(%)**				
Aspirin	23.4	20.5	23.9	0.009
ACE-inhibitors or ARB	27.7	21.2	28.7	<0.001
Beta-blockers	27.6	22.3	28.6	<0.001
Statin	19.0	13.2	20.0	<0.001

### Day-and night variation of onset of symptoms

**Fig 2:**
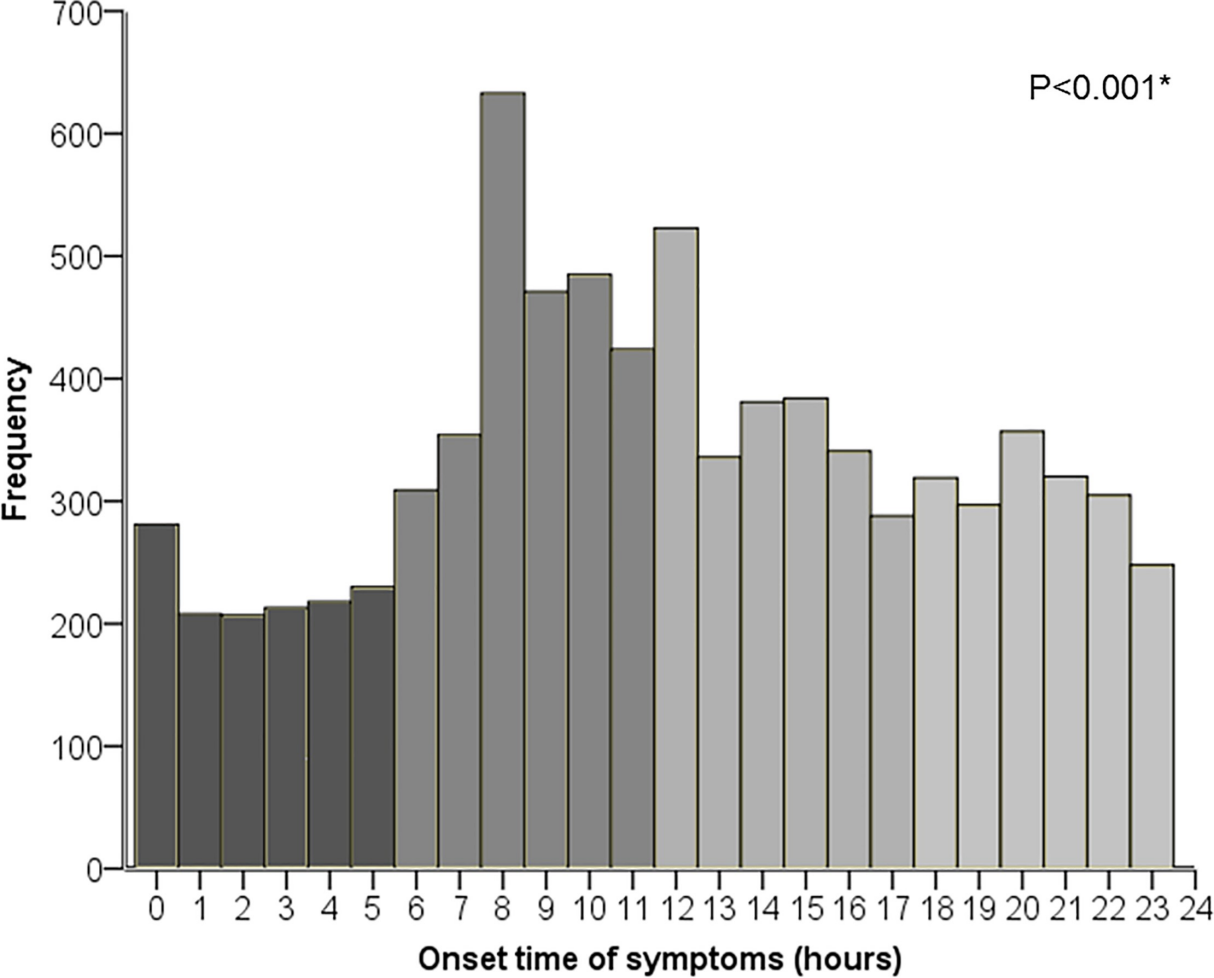
Onset of symptoms by hour. The different colors indicate time-periods in the following order; night, morning, afternoon and evening. A prominent increased risk for MINOCA was detected at 08.00 AM. *The presence of a circadian variation was confirmed with an overall likelihood ratio Chi-Square significance test, p<0.001.

The time (hour) for onset of symptoms was known in 8131 patients and the risk for MINOCA proved to be most common in the morning (IRR = 1.70, 95% CI [1.63–1.84]) and afternoon (IRR = 1.23, 95% CI [1.15–1.31]) with a peak at 08.00 AM (IRR = 2.25, 95% CI [1.96–2.59]) (Fig 2, Table 2). This finding was consistent for patients with STE-MINOCA, NSTE-MINOCA, women and men. Patients with age ≤65 years had an increased risk for MINOCA that was apparent between 06.00 AM and 09.00 PM and with a prominent peak at 08.00 AM, (IRR = 2.83, 95% CI [2.27–3.53]). Patients with age >65 years had an increased risk for MINOCA between 08.00 AM and 12.00 AM, with a peak at 08.00 AM (IRR = 1.90, 95% CI [1.59–2.29]).

**Table 2:** Results.

Onset time	Number	IRR (95%CI)	Onset time	Number	IRR (95%CI)
**Hour**	** **	** **	**Weekday**	** **	
00:00	281	reference	Saturday	1122	reference
01:00	208	0.74 (0.62–0.89)	Sunday	1210	1.08 (0.99–1.17)
02:00	207	0.74 (0.62–0.88)	Monday	1431	1.28 (1.18–1.38)
03:00	213	0.76 (0.63–0.91)	Tuesday	1253	1.12 (1.03–1.21)
04:00	218	0.78 (0.65–0.93)	Wednesday	1207	1.08 (0.99–1.17)
05:00	230	0.82 (0.69–0.97)	Thursday	1193	1.06 (0.98–1.15)
06:00	309	1.10 (0.94–1.29)	Friday	1218	1.09 (1.00–1.18)
07:00	354	1.26 (1.08–1.47)	**Months**		
08:00	633	2.25 (1.96–2.59)	January	720	reference
09:00	471	1.68 (1.45–1.94)	February	671	0.93 (0.84–1.04)
10:00	485	1.73 (1.49–2.00)	March	775	1.08 (0.97–1.91)
11:00	424	1.51 (1.30–1.75)	April	732	1.02 (0.92–1.13)
12:00	523	1.86 (1.61–2.15)	May	732	1.02(0.92–1.139
13:00	336	1.20 (1.02–1.40)	June	709	0.99 (0.89–1.09)
14:00	381	1.36 (1.16–1.58)	July	746	1.04(0.94–1.1)
15:00	384	1.37 1.17–1.59)	August	705	0.98 (0.88–1.09)
16:00	341	1.21 (1.04–1.42)	September	710	0.99 (0.89–1.09)
17:00	288	1.03 (0.87–1.21)	October	723	1.00 (0.91–1.11)
18:00	319	1.14 (0.97–1.33)	November	715	0.99 (0.90–1.10)
19:00	297	1.06 (0.90–1.24)	December	696	0.97 (0.87–1.07)
20:00	357	1.27 (1.09–1.49)	**Seasons**	** **	
21:00	320	1.14 (0.97–1.34)	Winter	2087	reference
22:00	305	1.09 (0.92–1.28)	Spring	2239	1.07 (1.01–1.14)
23:00	248	0.88 (0.74–1.05)	Summer	2160	1.04 (0.98–1.10)
**Time of day**	** **		Autum	2148	1.03 (0.97–1.09)
Night	1666	reference	**Holidays**		
Morning	2890	1.70 (1.63–1.84)	Christmas holidays	332	1.07 (0.85–1.13)
Afternoon	2049	1.23 (1.15-1-31)	Christmas	30	0.86 (0.55–1.34)
Evening	1527	0.917 (0.86–0.98)	Midsummer festivities	190	1.24 (1.00–1.54)

### Weekly variation in onset of symptoms

**Fig 3:**
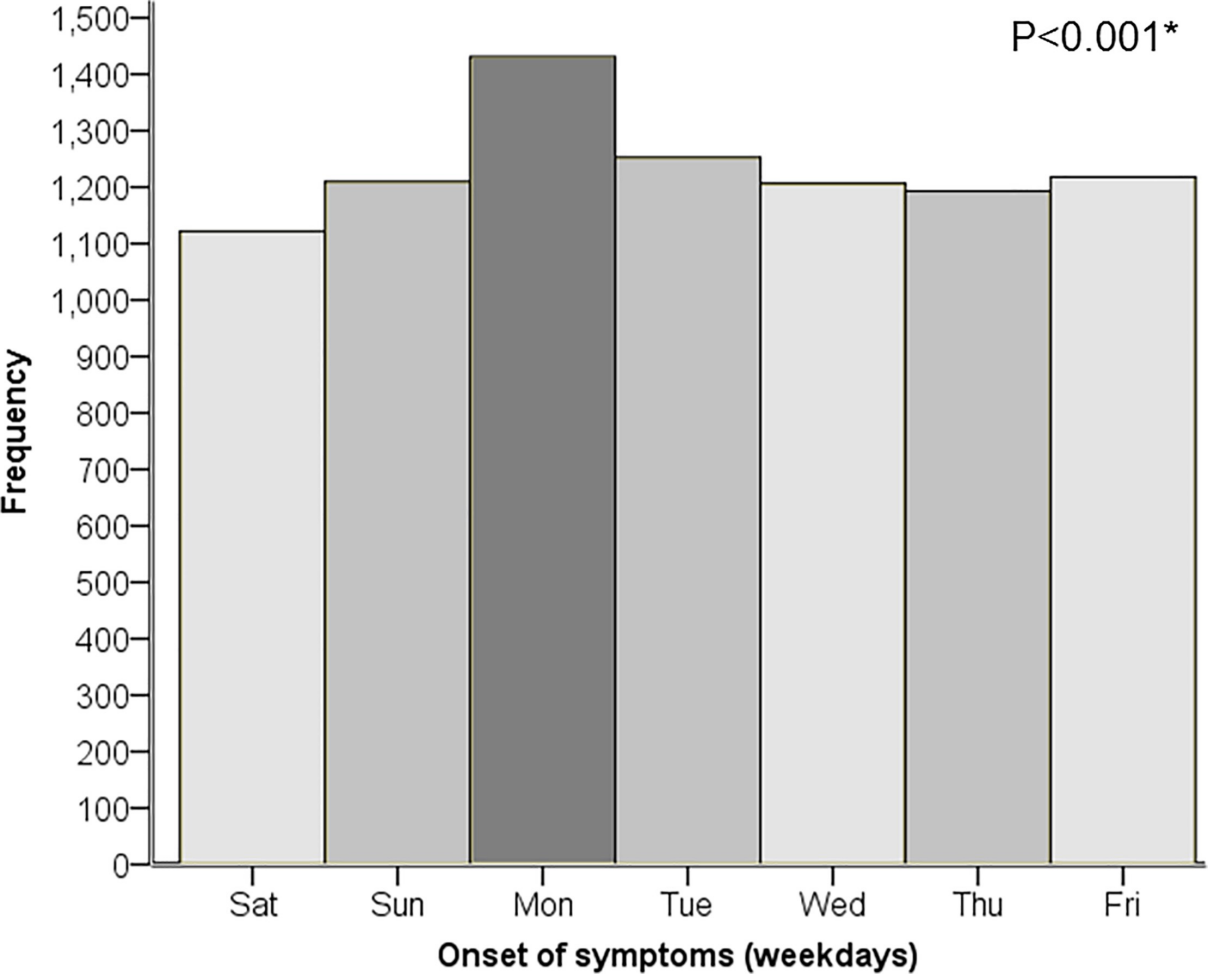
Onset of symptoms by weekday. A prominent increased risk for MINOCA was detected at Mondays. *The presence of a circaseptian variation was confirmed with an overall likelihood ratio Chi-Square significance test, p<0.001.

The weekday for the onset of symptoms was known in 8634 patients and a circaseptan variation in IRR of the onset of MINOCA was observed (Fig 3). The risk of MINOCA was higher on Mondays (IRR = 1.28, 95% CI [1.18–1.38]) and lowest in the weekends (Table 2). The circaseptan variation was observed with women, men, patients aged ≤65 years and aged >65 years, with NSTE-MINOCA but not with STE-MINOCA (IRR = 1.07, 95% CI [0.88–1.28]).

### Seasonal variation in onset of symptoms

**Fig 4:**
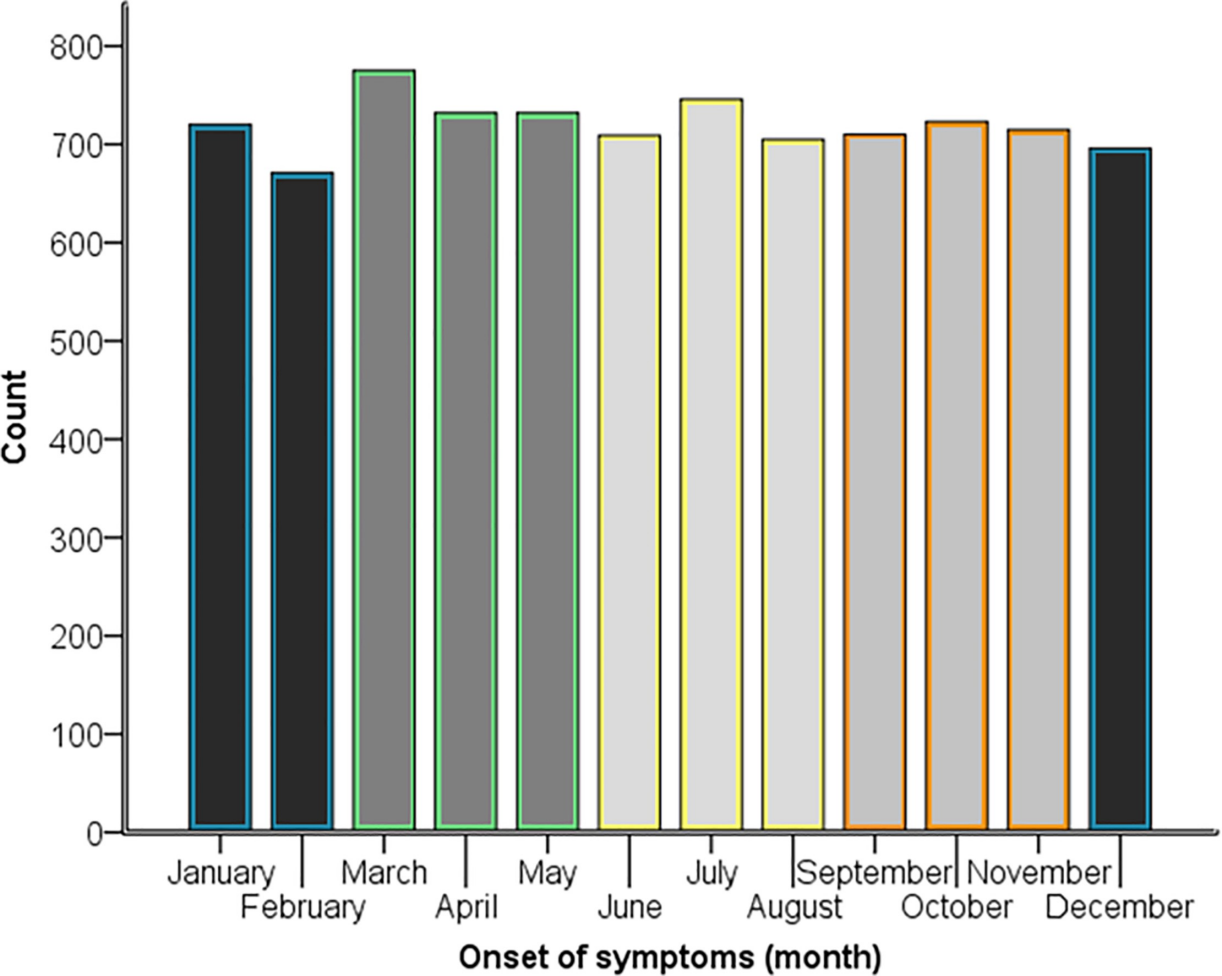
Onset of symptoms by month. The different colors indicate the seasons in the following order; two months of winter, spring, summer and 1 month of winter. *An overall likelihood ratio Chi-Square significance test excluded the presence of a monthly variation, p = 0.503 and a seasonal variation, p = 0.144.

The month of onset of symptoms of MINOCA was known for 8634 patients but no monthly variation was observed in the total group or in women, men, patients younger or older than 65 years or those with STE-MINOCA or NSTE-MINOCA (Table 2, Fig 4). When the year was divided into the four seasons, springtime yielded a 7.3% higher overall risk of MINOCA (IRR = 1.073, 95% CI [1.01–1.14]) (Table 2, Fig 4). The highest associated risk during spring was observed for patients ≤65 years, (IRR = 1.096, 95% CI [1.00–1.20]). However, when an overall likelihood ratio Chi-Square significance test was applied, the seasonal variation was not statistically significant (p = 0.144).

### Holidays

The risk for MINOCA during the Christmas and New Year holidays was similar to that two weeks preceding or following the holidays (IRR = 1.073, 95% CI [0.85–1.13]). Also, the risk for onset of MINOCA during Christmas Eve and Christmas Day was unchanged as compared to corresponding days one week prior and one week after Christmas (IRR = 0.85,7 95% CI [0.55–1.34]). These result were consistent for women, men, patients aged ≤65 or more than 65 and for STE-MINOCA and NSTE-MINOCA.

An increase in the risk for onset of symptoms of MINOCA was detected during the Swedish Midsummer holiday as compared to the weeks preceding and following the holiday (IRR = 1.24 95% CI [1.00–1.54]). This was also seen for men (IRR = 1.41 95% CI [1.00–1.99]) but not for women (IRR = 1.11 95% CI [0.84–1.48]), nor for patients aged ≤65 or >65 or those with STE-MINOCA or NSTE-MINOCA (Table 2). However, when we performed an overall likelihood ratio Chi-Square significance test, there was no statistically significant different risk for MINOCA during Midsummer, p = 0.110.

### Prognosis

As previously reported experienced a total of 2,147 patients (24.0%) at least one new MACE during a mean follow-up of 4.5 years [10, 14]. The total number of events during follow-up was; 1,254 deaths, 624 new MIs, 403 ischemic strokes and 580 hospitalizations with heart failure. Of the 1,165 deaths occurring after the first 30 days, only 497 (42.7%) were classified as cardiovascular deaths.

#### Short-term prognosis

During the index hospital stay 68 patients (0.7%) died and the one-month mortality rate was 1.0% (89 patients). The time (hour) of onset of symptoms was known for 74 patients (83%) and the day and month was known for 81 of the 89 patients who died within 30 days. We found no association between death within 30 days and the time of onset of symptoms, time of day, weekday, month or season when symptoms were detected.

#### Long-term prognosis -hour and time period

There was no association between all-cause mortality and hour or part of day of onset of symptoms. However, in univariate analysis, onset of symptoms during the night was associated with more MACEs (HR 1.17, 95% CI [1.01–1.34]) whereas start of symptoms in the afternoon resulted in fewer MACEs (HR 0.58, 95% CI [0.42–0.82]) at 1 pm and (HR 0.70, 95% CI [0.51–0.96]) at 4 pm. In a multivariate Cox regression model consisting of the time (hours or part of day) and previously known risk factors for MACE (gender, diabetes, age group, smoking and LDL-cholesterol group) time of onset of symptom lost association with MACE.

#### Long-term prognosis–weekday, month and season

There was no association between the weekday, month or season of onset of symptoms and all-cause mortality. Neither could any association between weekday or month and MACE be detected. A decreased risk of MACE was present for patients with MINOCA during February (HR 0.79 95% CI [0.63–0.99]), but no overall seasonal association with MACE could be detected.

## Discussion

This is the first large, nationwide, study investigating the presence of circadian, circaseptan and seasonal variations in the onset of MINOCA. With data from 9,092 unique MINOCA patients we were able to demonstrate a circadian onset of MINOCA, with a 70% increased risk for onset during the morning and a more than doubled risk at 8 AM. Furthermore, the onset of MINOCA was 27% higher on Mondays and considerably lower during the weekends. No monthly, seasonal or holiday related increased risk for MINOCA was detected.

### Circadian rhythm

The risk for MINOCA proved to be most common in the early morning with a peak at 8 AM. This finding is in concordance with previous findings from various MI populations [2–4, 6]. The morning triggering for both MINOCA and MI may be due to a synergistic effect of environmental and physiological factors, including increases in physical or mental stress [17] and circadian variations in cardiovascular, hormonal and metabolic functioning [2]. In our MINOCA population patients ≤65 years, most of which were employed, had an increased risk for MINOCA throughout the day while for the mostly retired patients aged >65, the increased risk for MINOCA was limited to the morning. The strong association between onset on Mondays for MINOCA patients in the present study and in previous studies of patients with all MI [6–8] suggests that work-related factors may be of importance. Mental stress is known to induce transient myocardial ischemia in a large proportion of patients with CAD [18]. Testing of mental stress–induced myocardial ischemia has indicated that ischemic responses are induced, not by extremely severe emotional stress but rather by behavioral challenges common in everyday workday such as mental arithmetic, public speaking tasks, problem solving tasks, cognitive tasks and reaction time tasks [18]. The hemodynamic responses to mental stress often involve increased heart rate and blood-pressure, and the stress-induced changes in the myocardium may be related to the increase in systemic vascular resistance, the coronary artery constriction and microvascular changes [18]. Furthermore, the presence of mental stress–induced myocardial ischemia is generally not related to the angiographic severity of the coronary artery disease but has been associated with adverse prognosis in patients with coronary artery disease [18, 19].

### Seasonal variation

The onset of MINOCA showed statistically significant monthly variation but when the year was divided into four seasons, springtime turned out to yield a borderline significant 7.3% higher overall risk of MINOCA compared to winter time. However when applying an overall likelihood ratio Chi-Square significance test, the seasonal variation was not statistically significant. Therefore, we conclude that there is no clear evidence of a seasonal variation in occurrence of MINOCA. This distinguishes MINOCA from MI, which as first described in 1937 and later confirmed in several studies, is more common during winters [20–22]. This and the dissimilar sex distribution indicate different pathophysiological mechanisms for MINOCA and MI. Environmental variables such as the sunlight have also proved to play an important role in the onset of MI in both hemispheres [20]. Previous studies have suggested an increased onset of MI during daylight hours [23], during periods of short sunlight duration [24] and a “summer shift” with decreased difference between numbers of diurnal and nocturnal MIs during the summer [20]. Air temperature, low atmospheric air pressure and high wind velocity have also been demonstrated to have a strong association with MI [24].

### Holidays

Contrary to our expectations no increased risk for MINOCA was detected during the Christmas days or during the two weeks including Christmas Eve, Christmas Day and New Year’s Eve. This finding differs with two recent studies on all Swedish MI patients demonstrating a significant increase of traditional MI-CAD during Christmas [6, 7] and New Year holidays [7]. No change in the incidence of MINOCA was nor noted during the Swedish Midsummer festivities, further indicating the difference between the pathophysiological mechanisms for MINOCA and MI, the latter having a strong association with the presence of sunlight [20, 24].

### Prognosis

In the present study we found no association between the time of onset of MINOCA and short- or long-term prognosis. This finding suggests that the underlying pathological mechanisms of MINOCA don’t vary between different onset times or periods even if the triggering mechanism may alternate. It also indicates that the quality of care of MINOCA patients is similar night and day, workdays and weekends.

Studies of the association between the onset of MI and prognosis have shown contradicting results. Some have, as the present study, not demonstrated any association between time of onset of MI and prognosis [3, 4] whereas others have demonstrated higher mortality after onset is in the morning [25] or during winter [9].

## Limitations

Our study has some limitations. Firstly, observational studies based on data from registries are not able to determine causality. Secondly, MINOCA patients form a heterogeneous group with several different underlying pathophysiological mechanisms such as plaque rupture, coronary artery spasm, coronary dissection, thrombosis with spontaneous thrombolysis, type 2 MI and clinically unrecognized myocarditis or Takotsubo syndrome [13]. Thirdly, any results of investigations after the initial hospitalization that may have changed the initial MINOCA diagnosis (e.g Cardiac Magnetic Resonance Imaging intracoronary imaging or computed tomography scan) are not registered in SWEDEHEART and therefore not possible to be considered. The knowledge about Takotsubo Syndrome was limited at the beginning of the study period and there may be a smaller number of Takotsubo Syndrome cases in the cohort, incorrectly diagnosed as having MI. Due to low number of adverse advents within 30 days of onset of MINOCA the present study has, due to low power, limited ability to detected associations between onset time and short- term prognosis. The limited number of onsets of symptoms during the holidays also limited the power to detect any holiday related associations.

## Conclusion

In this nationwide observational study of 9,092 patients with MINOCA a circadian and circaseptan variation in the onset of MINOCA was seen with higher risk early mornings and Mondays, similar to previous studies on all MI (mostly MI-CAD). However, during holidays were traditional MI increase, we did not see any increase for MINOCA.

As individuals over 65 years only had an increased risk during the morning while younger working individuals had an increased risk throughout the day, an additional work-stress related triggering factor may be present for the latter group. No association between onset time and short- or long term prognosis was detected which may suggest that the underlying pathological mechanisms of MINOCA don’t vary between different onset times even if the triggering mechanism may alternate. It also indicates health care with equal quality regardless of time of onset.
